# Long-term outcomes of patients with gastric adenoma in Korea: A retrospective observational study

**DOI:** 10.1097/MD.0000000000019553

**Published:** 2020-03-20

**Authors:** Tae Young Park, Su Jin Jeong, Tae Hyung Kim, Jin Lee, Jongha Park, Tae Oh Kim, Yong Eun Park

**Affiliations:** a Division of Gastroenterology, Department of Internal Medicine, Maryknoll Hospital; b Division of Gastroenterology, Department of Internal Medicine, Inje University School of Medicine, Haeundae Paik Hospital, Busan, Republic of Korea.

**Keywords:** endoscopic submucosal dissection, gastric neoplasm, intestinal metaplasia, lesion size, recurrence, risk factors

## Abstract

New endoscopic resection techniques are constantly being developed for gastric adenoma, which can be classified as low or high grade according to the Vienna classification. However, long-term data on gastric adenoma (e.g., removal or follow-up after resection via endoscopy) remain lacking.

We retrospectively analyzed 133 cases with gastric adenoma that underwent endoscopic resection from January 2010 to November 2018. We analyzed the risk factors and frequency of patients with synchronous and metachronous lesions after endoscopic resection for gastric adenoma and followed them for more than 2 years.

One hundred six (79.7%) and 27 patients (20.3%) received endoscopic resection (ER) once and more than twice, respectively. Compared with the initial endoscopic biopsy pathological results, the upgraded and downgraded histological discrepancy rates were 10.5% (n = 14) and 3.0% (n = 4) after resection, respectively. The mean time to synchronous/metachronous recurrence was 2.23 years. The average lesion size at first procedure was larger in the multiple ER group than in the single ER group (2.00 vs 1.10 cm; *P* = .040). Eleven (8.3%) and 16 patients (12.0%) had recurred synchronous and metachronous lesions, respectively. In the multivariate Cox analysis of the recurrence group, intestinal metaplasia (hazard ratio, 2.761; 95% confidence interval, 1.117–6.820; *P* = .028) and lesion size (hazard ratio, 1.607; 95% confidence interval, 1.082–2.385; *P* = .019) were independent factors for receiving endoscopic resection more than twice.

If patients have severe intestinal metaplasia or large size of lesion at endoscopic resection for gastric adenoma, periodic observation is necessary.

## 1 Introduction

Gastric adenomas are regarded as premalignant lesions that may develop into gastric cancer according to the dysplasia grade.^[1,2]^ Correa's cascade suggests that the mechanism underlying chronic gastric inflammation is chronic atrophic gastritis caused by *Helicobacter pylori* infection or other factors, leading to intestinal metaplasia (IM), gastric dysplasia, and gastric adenocarcinoma.^[3]^ In the revised Vienna classification,^[4]^ gastric epithelial neoplasia is classified under category 1 (negative for dysplasia) to 5 (submucosal invasion by carcinoma). Non-invasive low- and high-grade dysplasias (LGD and HGD) are classified under categories 3 and 4, respectively. HGD is considered a precancerous lesion, requiring removal via surgery or endoscopic submucosal dissection (ESD).^[5–7]^ Conversely, LGD is less likely to progress into gastric cancer than HGD; however, LGD treatment guidelines have not yet been established.^[7,8]^

Recently, the number of gastrointestinal endoscopies performed is increasing. Because of the high incidence of gastric cancer especially in Korea, the National Cancer Screening Program was initiated in 1999, which enforced performance of screening gastrointestinal endoscopy every 2 years for healthy individuals aged over 40 years. Since the start of this program, the incidence of gastric cancer slightly decreased from 43.6 per 100,000 individuals in 1999 to 35.8 per 100,000 in 2014; however, the proportion of non-epithelial tumor increased (from 0.8% to 1.4%) in Korea.^[9]^ Therefore, the diagnosis rate of gastric adenomas during endoscopy is increasing. However, histological discrepancies were reported between biopsy and resected specimens. When gastric adenomas are found, gastric cancer may already be present focally.^[10]^ Gastric adenomas diagnosed via endoscopic biopsy can be upgraded to invasive carcinomas after endoscopic resection (ER) (range, 4%–30%).^[11–13]^ Therefore, 1-piece or en bloc resection of gastric adenomas is necessary because it is possible to evaluate the appropriate treatment via histological assessment and reduce local recurrence. ER, including endoscopic mucosal resection (EMR) and ESD, is usually used for accurate diagnosis and treatment of gastric adenomas.^[14]^ ESD is preferred in patients with gastric adenoma in Korea and Japan because it can resect large and depressed lesions, which are difficult to resect via EMR.^[15–17]^

Several guidelines have been proposed for the treatment of HGD and LGD. The revised Vienna classification recommends endoscopic treatment for HGD and endoscopic treatment or follow-up for LGD.^[4]^ European guidelines recommend that endoscopic resection of LGD for accurate pathological diagnosis.^[18]^ Therefore, in some cases, such as LGD, it may be difficult to determine whether endoscopic resection is appropriate. In the case of ER for early gastric cancer, many studies have been done on the risk of recurrence and the recurrence rate after ER. However, there is a lack of research on how long endoscopic follow-up is necessary and how many metachronous lesions occur in gastric adenoma.

Several studies have reported the characteristics and prognosis of gastric adenomas compared with gastric cancer; only a few studies have investigated the risk factors of recurrence and long-term outcomes in gastric adenoma.^[12,14,17,19,20]^ We retrospectively analyzed the characteristics and risk factors of synchronous and metachronous recurrences, with a focus on gastric adenoma that underwent ER during their long-term follow-up.

## 2 Materials and methods

### 2.1 Patients

One thousand eighteen patients underwent ER for gastric adenoma at Inje University Haeundae Paik Hospital in Busan, Korea, between January 2010 and November 2018. Gastric adenoma was classified as low or high grade according to the revised Vienna classification.^[4]^ To evaluate the long-term follow-up outcomes, we excluded 889 patients owing to the following exclusion criteria:

(i) no endoscopic follow-up for at least 2 years
(ii) unavailable clinical data or records
(iii) history of gastric cancer surgery for advanced gastric cancer
(iv) no follow-up during the study period

A total of 116 patients who underwent ER for gastric adenoma between 2010 and 2016 and follow-up endoscopy between 2017 and 2018 were selected. However, to compare the number of ER cases performed in 1 patient (i.e., once vs twice or more), a total of 133 cases were retrospectively analyzed based on the number of ER cases received. This study was performed in accordance with the ethical guidelines of the 1975 Declaration of Helsinki and approved by our institutional review board.

### 2.2 Endoscopic and pathological findings

#### 2.2.1 Lesion locations

In cases of multiple lesions at the time of ER, only the more advanced dysplastic lesion was analyzed as the main lesion. If the lesions had the same grade of dysplasia, the largest lesion was included in the main lesion. The tumor location was divided into long and short axes. Long axis included the antrum, lower body, midbody, upper body, and cardia/fundus. Short axis included the anterior wall, posterior wall, lesser curvature, and greater curvature.

#### 2.2.2 Endoscopic findings

The underlying endoscopic findings were classified using the Kimura-Takemoto classification.^[21]^ Atrophic gastritis was defined when the mucosa of the antrum and body was thinned, and the submucosal blood vessels were well visible. IM was defined as the presence of white plaques or patches or discoloration of the mucosa (i.e., uniform white). Atrophy and IM were diagnosed on the basis of the assessment of the expert endoscopist. IM was also diagnosed on the basis of the pathological findings.

Endoscopic gross findings according to the Paris classification were simplified into 3 groups: polypoid/elevated (type 0–I), flat (types 0–IIa, 0–IIa+IIc, 0–IIb, 0–IIc, and 0–IIc+IIa), and depressed (type 0–III).^[22]^*H. pylori* infection was defined as either positive urease test or urea breath test findings or pathological confirmation.

#### 2.2.3 Endoscopic procedure

All gastric adenomas were removed endoscopically via EMR with pre-cutting (EMR-P) or ESD. The shapes and margins of the gastric lesions were identified using single-channel endoscopy (GIF H260, Olympus, Tokyo, Japan) before ER. Using argon plasma coagulation, the lesion boundary was marked by dotted lines. Isotonic saline with dilute epinephrine (1:10,000) was injected to the submucosal layer to elevate the lesion. For EMR-P or ESD, a circumferential incision was created around the lesion, and the lesion was dissected using a snare (EMR-P) or an insulated tipped knife (ESD).

En bloc resection was defined as resection of a single piece and piecemeal resection as that of multiple pieces of specimen. Complete resection was defined as successful en bloc resection with histologically free lateral and vertical margins.

#### 2.2.4 Pathological findings of specimen

The specimen was diagnosed by 2 pathologists in our hospital. The pathologists were experts with more than 5 years of experience. When the biopsy was done at a local clinic hospital, the tissue was taken to our hospital and re-diagnosed by the pathologists in our hospital.

The size of lesions and specimens was based on the results of measurements made by a ruler in the pathology department. The specimen size was defined as the longest measured size of the specimen after ER. The lesion size was defined as the longest pathologically measured length. An upgraded histological discrepancy in LGD was defined as histologically diagnosed HGD (category 4) or invasive gastric cancer (category 5) in the endoscopically resected specimens; a downgraded histological discrepancy was defined as the final histology showing negative (category 1) or indefinite findings for neoplasia or dysplasia (category 2). In HGD, an upgraded pathology was defined as invasive gastric cancer (category 5) in the endoscopically resected specimens and a downgraded pathology as LGD (category 3) or the presence of negative (category 1) or indefinite dysplasia (category 2) findings.

#### 2.2.5 Follow-up after ER

All patients underwent the first follow-up endoscopic examination 3 months after endoscopic resection. Follow-up endoscopic examination was performed at 3, 6, 12, and every 12 months thereafter. However, in few cases, the examination was not performed at 6 months, depending on the expert endoscopist's preference.

#### 2.2.6 Recurrence

Synchronous lesions were defined as newly detected neoplasm within 6 months after ER of the primary gastric adenoma. Metachronous lesions were defined as newly discovered lesions at another site 6 months after ER of the primary gastric adenoma.^[23]^

### 2.3 Statistical analysis

Variables were expressed as medians [interquartile ranges (IQRs)] or n (%). The baseline characteristics were compared using independent Student *t* test or Mann–Whitney *U* test for continuous variables and the χ^2^ test or Fisher exact test for categorical variables, as appropriate. We compared the baseline characteristics of the patients who received ER once and more than twice and analyzed the differences according to the adenoma type and presence of recurrence. Independent predictors of recurrence among the endoscopic therapy patients were analyzed using Cox regression analyses. Hazard ratios (HRs) and the corresponding 95% confidence intervals (CIs) were calculated. Additionally, the overall cumulative risk rates of recurrence following underlying gastric mucosal description were analyzed using the Kaplan–Meier method and compared using the log-rank test. Data analysis was performed using SPSS (version 25.0, IBM Corp., Armonk, NY). *P*-values of <.05 were considered statistically significant.

## 3 Results

### 3.1 ER conducted once vs more than twice

Between January 2010 and November 2018, 116 patients (133 cases) were diagnosed with gastric adenomas and underwent ER. All patients underwent a first follow-up endoscopic examination 3 months after ER. In most patients, follow-up endoscopy was scheduled at 3, 6, and 12 months after ER, and annually thereafter. The average number of follow-up endoscopic examinations performed on patients was 5.06 (standard deviation, 2.33) times. Twenty-seven of them (20.3%) had 1 or more recurrences, with the greatest number of recurrences occurring 3 times. A total of 133 gastric adenomas were analyzed, and the patients’ baseline characteristics are shown in Table 1. There were 79 men (59.4%) and 54 women (40.6%), with an average age of 63 (IQR, 56–70) years. Seventy-seven patients (57.9%) with adenoma underwent endoscopy for screening at a local clinic hospital before ER.

**Table 1:**
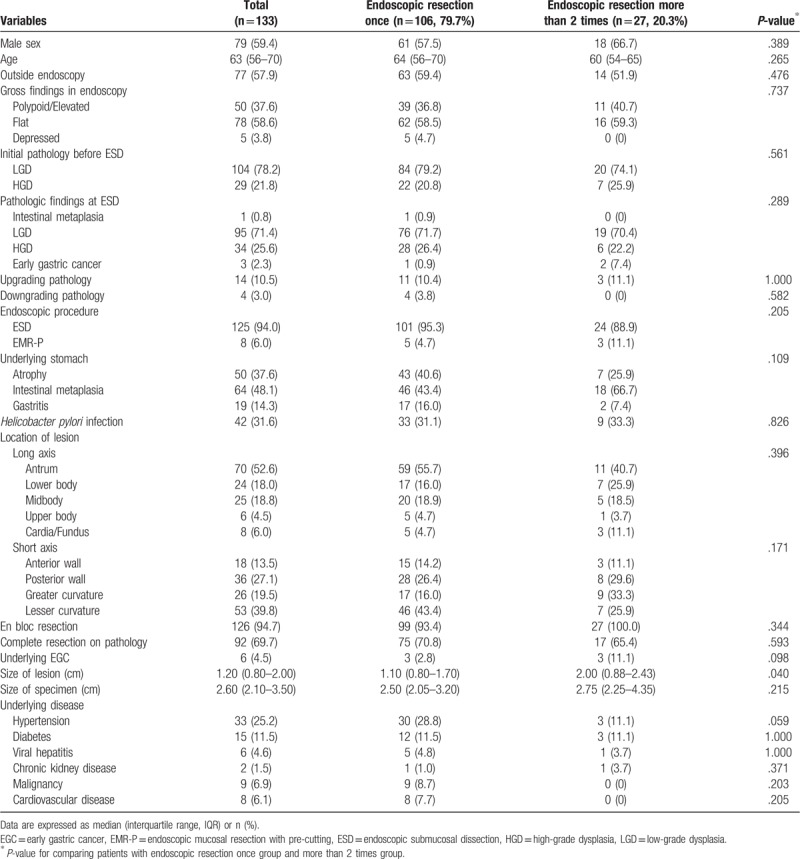
Baseline characteristics of study subjects.

The most common type of endoscopic gross finding was flat lesions in 78 adenomas (58.6%). LGD was the most common pathological finding in both groups (79.2% and 74.1%). A pathological discrepancy between endoscopic forceps biopsy and ER was observed. The pathological findings in forceps biopsy were LGD in 104 adenomas (78.2%) and HGD in 29 adenomas (21.8%). However, those after ER were LGD in 95 adenomas (71.4%) and HGD in 34 adenomas (25.6%). The upgraded and downgraded histological discrepancy rates were 10.5% (n = 14) and 3.0% (n = 4), respectively.

The endoscopic procedure was ESD in 94.0% and EMR-P in 6.0%. IM accounted for almost half of the underlying gastric conditions (48.1%); the *H. pylori* infection rate was 31.6%. The lesions were mainly located at the antrum (long axis; 52.6%) and lesser curvature (short axis; 39.8%).

The en bloc resection rate was 94.7% (n = 126); however, the complete resection rate on pathology was lower (69.7%; n = 92). The average lesion size was 1.20 (IQR, 0.80–2.00) cm, and the average specimen size was 2.60 (IQR, 2.10–3.50) cm. The average lesion size (1.10 [IQR, 0.80–1.70] cm vs 2.00 [IQR, 0.88–2.43] cm; *P* = .040) was significantly larger in the multiple ER group than in the single ER group (Table 1).

### 3.2 LGD and HGD

According to the pathological type of the initial gastric adenoma, HGD group had a higher proportion of men than LGD group (79.3% vs 53.8%; *P* = .014) (Table 2). Further, 10.3% of the HGDs were diagnosed as early gastric cancer (EGC) after ER owing to upgraded histological discrepancies. The downgraded histological discrepancy rate for HGD was also 10.3%, which was higher than that for LGD (*P* = .032). In addition, the size of specimen in HGD group was larger than that of the LGD group (*P* = .009). All HGDs were removed via ESD. The recurrence rate was 19.2% (n = 20/104) in LGD and 24.1% (n = 7/29) in HGD. In other items, there was no significant difference between HGD and LGD (*P*>.05) (Table 2).

**Table 2:**
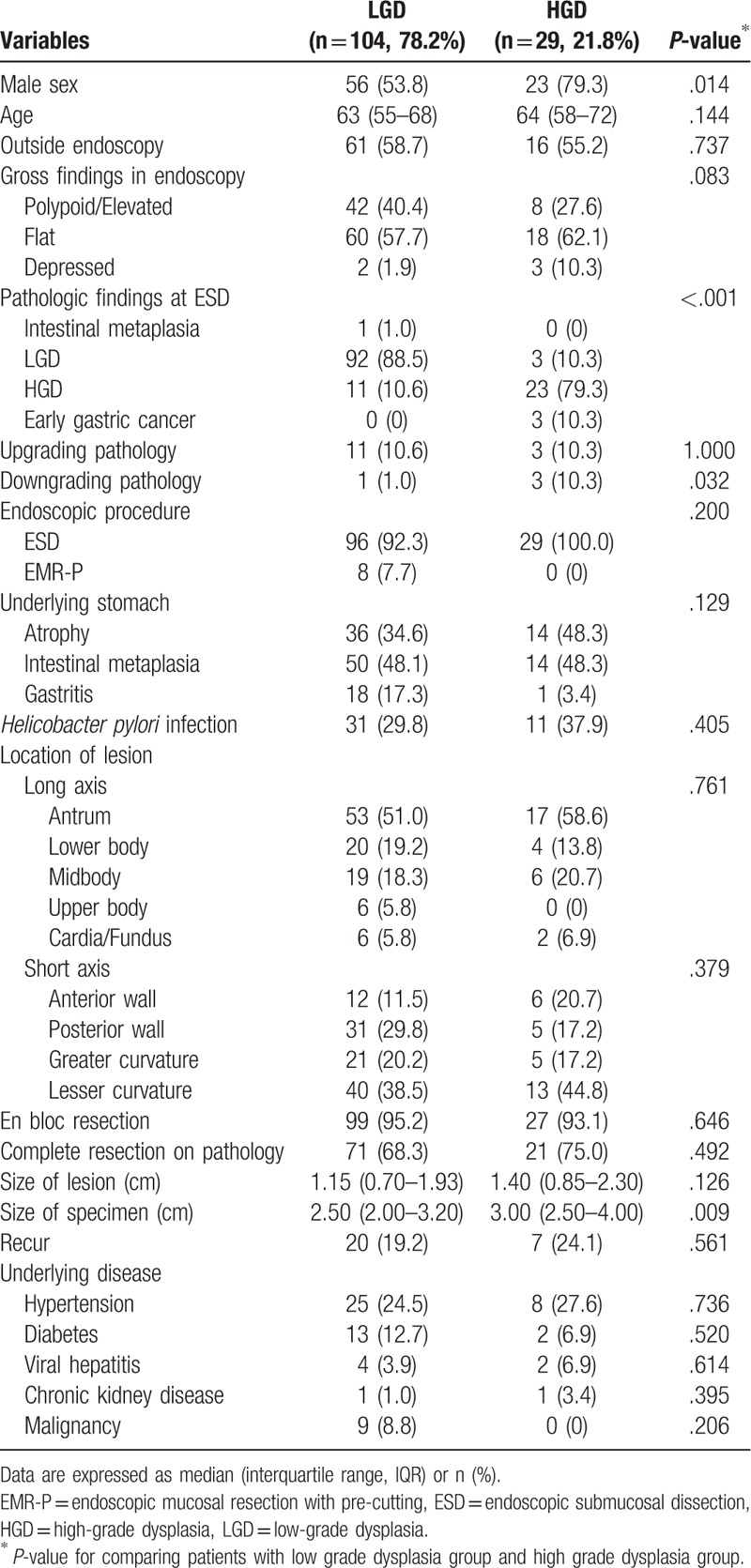
Baseline characteristics of study subjects according to pathologic type of initial gastric adenoma.

### 3.3 Recurrence: synchronous and metachronous neoplasms

During the study period, 27 gastric adenomas (20.3%) were identified as synchronous and metachronous neoplasms. There were 3 recurrences in 1 patient and 1 recurrence in all other patients. No local recurrence was observed in the ESD site and only synchronous/metachronous recurrence in the other site was analyzed. The patients’ baseline characteristics are shown in Table 3. There were 11 cases of synchronous (40.7%) and 16 cases of metachronous neoplasms (59.3%): 19 cases (70.4%) in LGD, 6 cases (22.2%) in HGD, and 2 cases (7.4%) in EGC. Among the initial pathological findings at the first ER, HGD was more frequent pathological finding in the metachronous recurrence group compared with that in the synchronous group (LGD [synchronous lesion, 81.8% vs metachronous lesion, 62.5%], HGD [0.0% vs 37.5%], EGC [18.2% vs 0.0%]; *P* = .016). However, there was no significant difference among the pathological findings at recurrence, such as LGD, HGD, and EGC, between the synchronous and metachronous recurring lesions (*P* > .05) (Table 3). Additionally, there was no significant difference in sex and age between the synchronous and metachronous lesion groups (*P* > .05). The most common type of endoscopic gross finding was also flat lesion in both groups (synchronous lesion, 63.6% vs metachronous lesion, 56.3%). The metachronous lesions tended to have more atrophic changes (18.2% vs 31.3%) than the synchronous lesions; however, IM (72.7% vs 62.5%) and gastritis (9.1% vs 6.3%) were similar compared with the synchronous lesions (*P* > .05). *H. pylori* infections were also more common in the metachronous lesions, although the difference was not significant (18.2% vs 43.8%; *P* = .231). Both synchronous and metachronous lesions were mainly located at the antrum (27.3% vs 50.0%) and greater curvature (36.4% vs 31.3%) as solitary lesions. The en bloc resection rate was 100.0%; however, the pathological complete resection rate for the synchronous lesions was lower than those for the overall mean and metachronous lesions (60.0% vs 68.8%; *P* = .692).

**Table 3:**
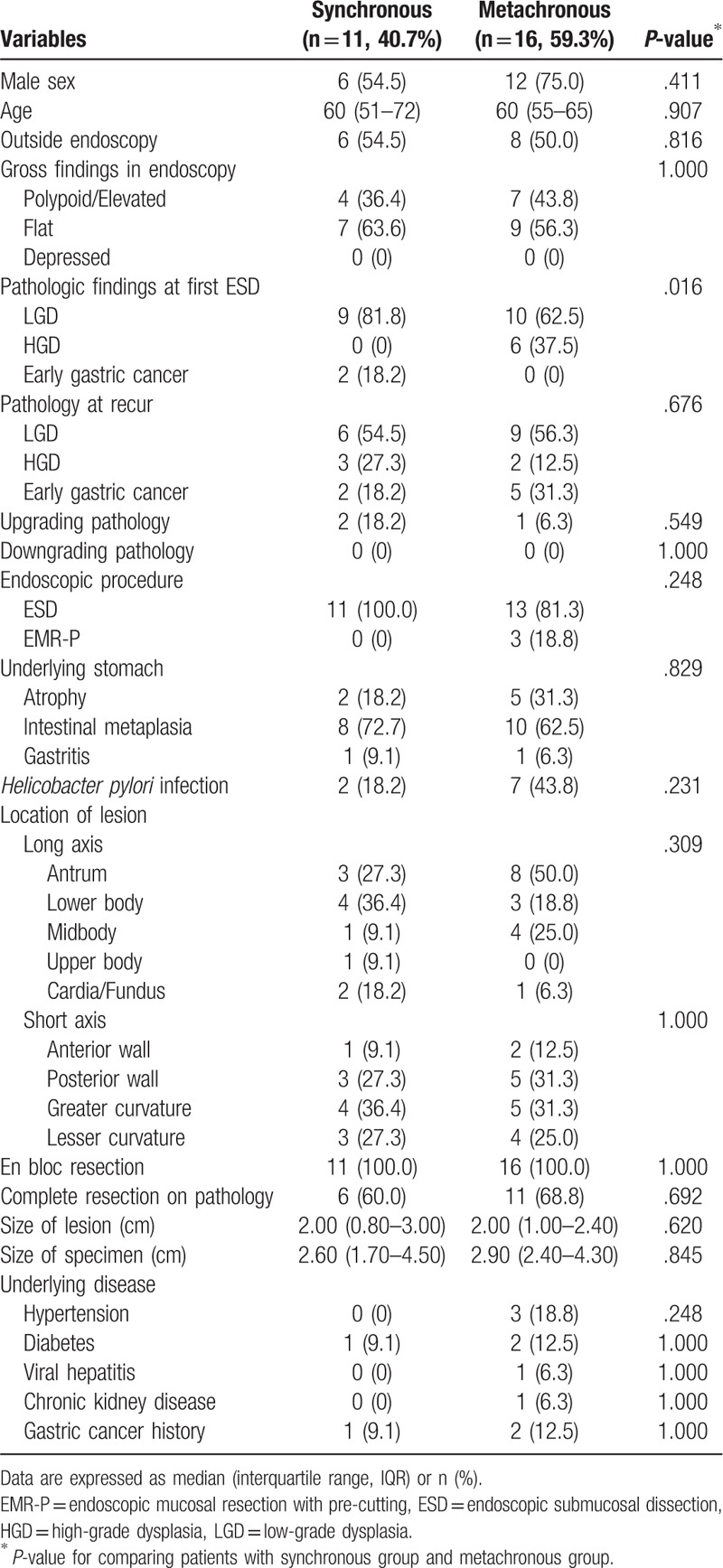
Baseline characteristics of study subjects according to synchronous and metachronous lesion in redo-procedure patients.

### 3.4 Risk factors of tumor recurrence after ER

**Figure 1:**
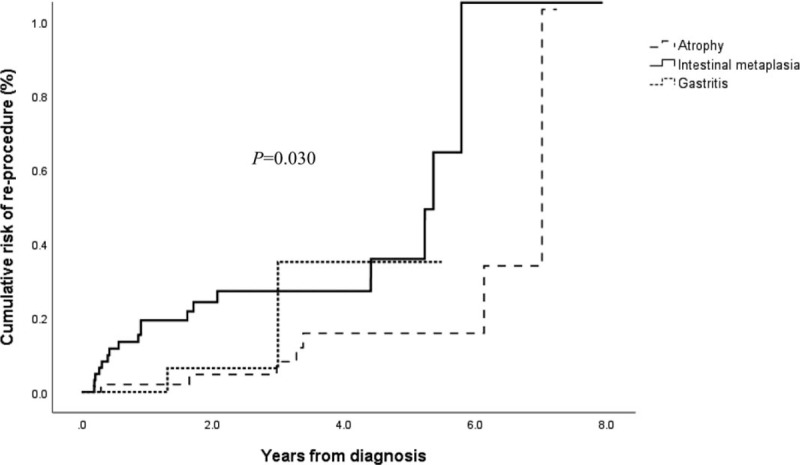
Cumulative recurrence rates following underlying gastric mucosal description (Kaplan–Meir graph).

Univariate and multivariate analyses were performed to determine the risk factors of recurrence after ER (Table 4). In the univariate analysis and multivariate analysis, IM (HR, 3.023; 95% CI, 1.233–7.407; *P* *=* .016) and lesion size (HR, 1.746; 95% CI, 1.166–2.615; *P* *=* .007) were found to be significant factors for recurrence. Of the variables, IM and lesion size significantly increased the risk in the multivariate analysis. The adjusted HR for IM was 2.761 (95% CI, 1.117–6.820; *P* = .028), and that for the lesion size was 1.607 (95% CI, 1.082–2.385; *P* = .019). The initial pathological findings before resection were not significant risk factors of recurrence (*P* *=* .809). The median duration for recurrence was 2.23 years. Furthermore, there was a significant difference in the adenoma recurrence rate according to the presence of gastric mucosal atrophy, IM, and gastritis in the log-rank curve (*P* = .030) (Fig. 1).

**Table 4:**
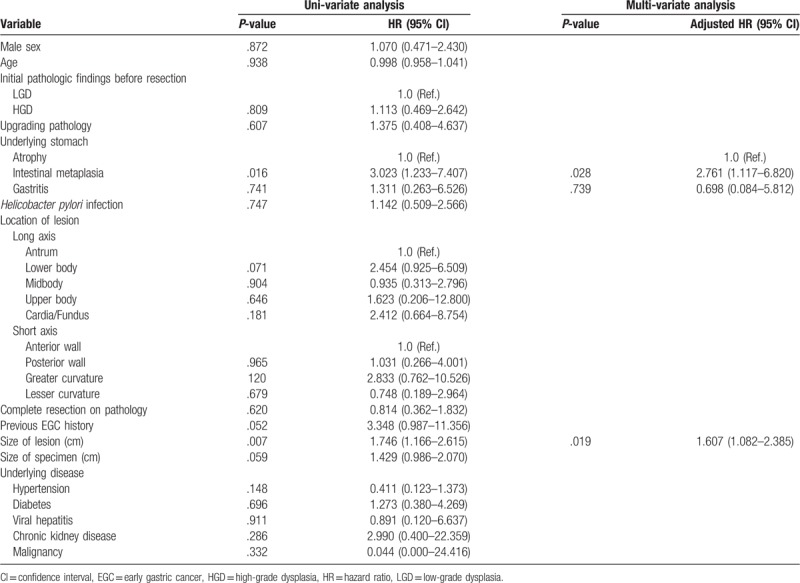
Risk factors of recur (Cox).

## 4 Discussion

This study aimed to evaluate the characteristics and long-term outcomes of gastric adenomas after ER. The characteristics of gastric adenomas and risk factors of recurrence after ER have been reported. However, previous studies^[7,24,25]^ have analyzed gastric adenomas with EGC; conversely, this study only included gastric adenomas. Herein, the median duration of recurrence was 2.23 years, and adenomas could recur as cancers (7/133, 5.26%); our recurrence rate was 20.3%. Further, IM and lesion size significantly increased the recurrence risk.

The incidence of synchronous or metachronous neoplasm after ER was 3.0 to 20.9%.^[7,26,27]^ One study reported the frequency of metachronous neoplasm according to the gastric adenoma grade, that is, 2.3% in LGD and 8.3% in HGD.^[28]^ In this study, the incidence of synchronous and metachronous lesions was 8.3% and 12.0% after ER, respectively. The incidence of metachronous neoplasm according to the gastric adenoma grade was 9.6% (10/104) for LGD and 20.7% (6/29) for HGD. This is similar to or slightly higher than the results of a previous study.^[28]^

Several studies have investigated the risk factors of recurrence after ER for gastric neoplasm. Severe atrophy and IM were reported as risk factors of synchronous and metachronous gastric neoplasms after ER for gastric adenoma.^[29]^ IM was also identified as a significant risk factor in this study. This is consistent with the findings of previous studies because IM is a precursor lesion of gastric adenoma and adenocarcinoma. It is the conversion of epithelium into other types of epithelium similar to that found in the intestines.^[30]^ Shichijo et al reported that the cumulative 5-year incidence of gastric cancer in IM was 3.2%.^[31]^ Therefore, the residual gastric mucosa with IM can increase the risk of tumor recurrence after ER.

Some studies have investigated the relationship between the gastric lesion size and gastric neoplasm recurrence. Most metachronous gastric adenocarcinomas and HGD frequently recurred when the lesion was larger than 2 cm in size.^[32]^ Similarly, in our study also showed that the gastric neoplasm size was significantly associated with increased recurrence risk.

The recurrence rates after ER among LGD, HGD, and EGC did not differ significantly.^[28]^ Similarly, our study showed that the initial pathology did not affect the recurrence. Further, a history of EGC also did not increase the recurrence risk. Both synchronous and metachronous lesions could occur if LGD is present after the first ESD. Cancer may develop as metachronous lesions even if diagnosed as gastric adenoma in the first ESD. In this study, among the patients with metachronous recurring lesion, the proportion of cases recurred as EGC was 31.3% (5/16), which was relatively high compared with 18.2% for the synchronous recurring lesions.

Our study showed the characteristics of the patients with adenoma who underwent ER, including the biopsy discrepancy and ESD pathology. Further, 10.6% of the lesions diagnosed as LGD in forceps biopsy were diagnosed as HGD or EGC after ESD; 10.3% of HGDs diagnosed in forceps biopsy were diagnosed as cancer after ESD, which is similar to previous results.^[17]^ Unlike previous studies, we analyzed only gastric adenomas and conducted long-term follow-up after ER from 2010 to 2018. Therefore, we determined the risk factors of gastric adenoma recurrence and mean duration of the recurrence. More careful endoscopic observation is needed for patients with recurrent risk factors, and a sufficient endoscopic follow-up period is required.

However, our study has several limitations. First, this was a retrospective study conducted in a single center; thus, selection bias may occur. The patients did not have the same endoscopic follow-up period owing to the different preferences of the practitioner. Second, we did not perform screening endoscopy again for all cases, particularly in cases wherein the gastric adenoma was found in a local clinic hospital, which were then transferred to the ER of our hospital. Thus, the possibility that some gastric adenomas already existed cannot be ruled out. However, we examined the entire stomach at the same time as ER to identify other lesions, there was no significant effect on the outcome. Third, all histological findings were not confirmed by the same pathologist. Some patients who underwent biopsy in a local clinic hospital did not bring the tissue slide but only pathological readings. Therefore, there may be discrepancy in some pathological readings.

There is no clear guideline regarding the endoscopic surveillance period after ER of gastric adenomas. In EGC, the average time to metachronous gastric cancer after ER was 3.1 (range, 1–8.6) years.^[33]^ Herein, the median follow-up period of the patients with metachronous lesions was 2098 (range, 1290–2539) days (more than 5 years after ER).

## 5 Conclusion

In conclusion, synchronous/metachronous tumors may occur in any patient, including those with LGD and HGD, after ER of gastric adenoma; thus, it is necessary to follow up patients at a high risk carefully. The recurrence risk after ER of gastric adenoma may be relatively high when there is severe IM and large lesion.

## Author contributions

**Guarantor of the article:** Yong Eun Park

**Jin Lee:** study concept and design; critical revision of the manuscript for important intellectual content.

**Jongha Park:** study concept and design; critical revision of the manuscript for important intellectual content.

**Su Jin Jeong:** study concept and design; critical revision of the manuscript for important intellectual content.

**Tae Hyung Kim:** study concept and design; critical revision of the manuscript for important intellectual content.

**Tae Oh Kim:** study concept and design; critical revision of the manuscript for important intellectual content.

**Tae Young Park:** acquisition of data; analysis and interpretation of data; drafting of the manuscript.

**Yong Eun Park:** acquisition of data; study concept and design; critical revision of the manuscript for important intellectual content.

All authors approved the final version of the article, including the authorship list.

Yong Eun Park orcid: 0000-0003-4274-8204.
